# Impact of Catastrophic Health Expenditures on Chinese Household Consumption

**DOI:** 10.3389/fpubh.2021.646494

**Published:** 2021-11-10

**Authors:** Ning Wei, Wenhao Huang, Lü-lin Zhou

**Affiliations:** ^1^School of Management, Jiangsu University, Zhenjiang, China; ^2^School of Management, Xuzhou Medical University, Xuzhou, China

**Keywords:** CHE, propensity score matching, China, household, consumption

## Abstract

China has built a social medical insurance system that covers the entire population so as to reduce the impact of diseases on individuals and families. Although the decline in the incidence of catastrophic health expenditures (CHEs) in China is encouraging, this issue remains important. On the basis of considering selectivity bias and heterogeneity, we applied propensity score matching (PSM) to analyze the 2018 data from the China Family Panel Studies. We assigned CHE households and non-CHE households to the treatment group and the control group, respectively, and used non-random data to simulate a randomized trial to investigate the impact of CHE on household consumption in China. The results of this study indicate that, when the threshold is set at 40%, the consumption of households experiencing CHEs (CHE household) is significantly lower than that of households not experiencing CHEs (non-CHE households) and that CHEs have a significant negative impact on other household consumption and a significant impact on the household property and debt. This effect still exists when the threshold is set lower, with household essential consumption most affected. The occurrence of CHEs leads to a reduction in household consumption and a significantly worsening financial situation for the CHE households, impacting the basic quality of life of the families. Therefore, it is necessary to further reform the medical and health system to reduce the high medical expenses.

## Introduction

Catastrophic health expenditures (CHEs) affect social welfare around the world (1). At the same time, CHEs may force families to reduce necessary consumption or fall into poverty. It is an important indicator for assessing the protection of household finances provided by health care systems (2, 3). As a type of household consumption, CHEs are substantially different from other types of household consumption. The most important manifestation is that medical expenses are consumption decisions that family members are forced to make when faced with medical treatment. Given the budget constraints determined by household income and property, families may have to reduce certain expenditures and consumption to pay CHEs.

In 1998, China officially established a social medical insurance system. After more than two decades of development, the social medical insurance system of China has been continuously improved, and the proportion of costs reimbursed has been steadily increasing based on the basic realization of full coverage of medical costs of the residents. However, although medical insurance provides reimbursements, residents are still responsible for a certain amount of out-of-pocket expenses. Therefore, the encroachment and crowding-out effect of medical expenses on other household consumption seems inevitable. Against the backdrop that China is the largest developing economy in the world, improving medical services coincides with challenges regarding financial protection (4). To this end, the Chinese government further established a nationwide system of critical illness insurance between 2012 and 2015. In 2016, the Chinese government began to integrate two systems, Urban Residents Basic Medical Insurance (URBMI) and New Rural Cooperative Medical Scheme (NCMS), to establish a unified basic medical insurance system for urban and rural residents. By gradually improving system construction, medical expenses incurred by families due to critical illness can be further reduced (5). In October 2017, General Secretary Xi Jinping stated at the 19th National Congress of the Chinese Communist Party that it is necessary to strengthen the social security system and improve the unified basic critical illness insurance system for urban and rural residents.

With continuous advancements in medical insurance reform in China, there has been a growing number of studies on household CHEs. Among them, a comparative analysis of the incidence of CHEs during the implementation of the Integration of Urban and Rural Medical Insurance Scheme (IURMIS) was performed using 2013 survey data, and the results showed that the incidence of CHEs in areas implementing the IURMIS (13.68%) was lower than that in areas that did not implement the IURMIS (13.87%). IURMIS of China is still in the exploratory stage. Therefore, even if a positive impact on low-income residents is confirmed, the impact will be limited (6). With the medical insurance system of China entering the postreform era, a study on the trends in CHEs and poverty in China after the healthcare system reform was conducted using nationally representative survey data. The results showed that between 2010 and 2016, the incidence of CHEs dropped from 19.37 to 15.11%, and the incidence of health poverty decreased from 7.39 to 5.14% (7). Another study using different survey data showed that, overall, the incidence of CHEs in Chinese households showed a declining trend from 2010 (12.57%) to 2016 (8.94%) and that the incidence of CHEs in the poorest families declined faster than that in the wealthiest families (8). These results indicate that the healthcare reform of China has made progress in reducing the occurrence of CHEs but that the incidence of CHEs in China is concentrated among poorer families, and the trends for this inequality have not changed significantly. In addition, different types of medical insurance schemes targeting different populations have significantly different financial protection effects against CHEs (9). The above studies show that, despite the encouraging decline in the incidence of household CHEs in China, this issue is still important because the burden of CHEs in Chinese households is still substantial and because increased population aging and slowing economic growth may generate pressure on the social medical insurance system of China. Therefore, we need to further study CHE-related issues in China, in which the impact of CHEs on household consumption and the corresponding strategies are important to address.

The contributions of this study are mainly reflected in three aspects. First, most of the previous studies discussed the incidence of household CHEs or the distribution of out-of-pocket payments among different families and did not investigate the effect of CHEs on non-medical consumption, especially household consumption. Second, in addition to affecting household basic consumption, CHEs may have a certain impact on the financial situation of a family, which is addressed in this study. Third, after many years of the social medical insurance system of China reform, the policy effects need to be evaluated using the latest data.

## Literature Review

The severity of an illness or medical expenses are not necessary conditions for the occurrence of CHEs. The existence of CHEs depends on two variables: out-of-pocket payments by a household and the total expenditure of the household (10). Conceptually, CHEs occur when the household out-of-pocket expenses trigger poverty or affect family sustainability. Therefore, it is important to know the ratio of medical expenses to total household expenses. However, there is no consensus on how to determine the threshold for CHEs (11, 12). There are three thresholds reported in the published studies. The first study sets the threshold at 10% when using household expenses as the denominator (13). The second study sets the threshold at 40%, which is commonly used for studies in developing countries (14). The third study sets the threshold within the range of 10–40% to show the differences among different countries under different socioeconomic conditions (13).

The existing studies have shown that CHEs may force families to reduce expenses other than medical expenses, such as food and education expenses. A study targeting rural areas in China found that for every 100 RMB increase in the per capita medical expenses in a household, the per capita food consumption expenses of a household decreased by 0.67%; for households within the lowest 25% income bracket, food expenses were reduced by up to 4.1% (15). Leive and Xu (12), based on studies of six African countries, found that when households faced CHEs, food consumption increased while other consumption declined, indicating that families first protected their food consumption needs (16). However, when the family medical expenditure to total expenditure ratio exceeds 40%, the have a significant impact on different household consumption items, indicating that there are differences in the impact of CHEs on different household consumption items; furthermore, there is a different relationship between the severity of CHEs and food consumption. A study by Kim and Yang in South Korea found that when CHEs occur, to avoid financial ruin caused by potential excessive medical expenses, families must reduce the consumption of other items (17).

In addition to CHEs, other factors that determine household consumption and expenditures also include household income, household size, household composition, and commodity price. Household income is one of the important factors affecting household consumption, and the amount, structure, and fluctuation of household income all determine consumption (18). Commodity price contains factors that many households cannot control. For example, families living in urban or rural areas may be unable to change the household daily consumption expenses (19). In addition, the household size and composition have an impact on household consumption expenditures. The larger the household size is, the more shared items in that household. Therefore, the expansion of household size does not require a corresponding increase in consumption expenditures to maintain the same living standards (20). Taking food consumption as an example, the larger the household size is, the smaller the per capita food consumption (21). In terms of the composition of family members, the sex of family members, the number of elderly people, race, and region all have different effects on family travel consumption patterns (22). When the elderly population in a household accounts for a relatively high proportion of all household members, the food consumption expenditures of the household are lower. This occurs because when the elderly individual enters the retirement stage, their production in the household increases, and thus, the food consumption expenditures in the household decrease (23).

In summary, household income, size, and composition are all factors that affect household consumption expenditures. These factors may also affect the occurrence of CHEs. That is, the difference in expenditures between households experiencing CHEs (CHE households) and households not experiencing CHEs (non-CHE households) may be due to the different characteristics of these two types of families, rather than simply attributed to CHEs. Therefore, to analyze the effect of CHEs on household basic consumption expenditures, it is necessary to fully consider the differences between different families.

## Materials and Methods

### Data Source

The data used in this study are from the China Family Panel Studies (CFPS) in 2018. The CFPS is a nationwide survey organized and conducted by the Institute of Social Science Survey of Peking University, Beijing, China. For the CFPS, data were collected on individuals, families, and communities through tracking to reflect changes in the society, economy, population, education, and health of China. The CFPS samples covered 25 provinces/cities/autonomous regions in China, and all the family members in the samples were included in the survey. In this study, we used the household questionnaire in the CFPS and used family as the sample to investigate the effect of CHEs on family consumption. A total of 14,137 valid household samples were obtained through data processing.

### Description of Variables

#### Catastrophic Health Expenditure

The data obtained from the CFPS household questionnaire that addressed family medical expenses in the past 12 months “excluded expenses already reimbursed and expected to be reimbursed but included expenses paid with borrowed money or paid by relatives and friends,” i.e., out-of-pocket payments. We used out-of-pocket payments as the numerator and annual total household consumption expenditure as the denominator to calculate the CHE. We used 40% as the CHE threshold (11, 14), a value commonly used in studies in developing countries, to distinguish whether CHEs occurred in families, and then, we divided all households into a CHE group and a non-CHE group. On this basis, we also analyzed the impact of CHEs on household consumption when the CHE threshold ranged from 10 to 40% (13).

#### Household Consumption Expenditures

Based on the classification standards in the *China Statistical Yearbook* and the consumption items included in the CFPS survey, we excluded medical expenditures and divided household consumption expenditures into seven major categories: (1) food expenditures, such as household food expenses and the purchases of snacks, beverages, tobacco, and alcohol; (2) clothing, footwear, and hat expenses, including tops, pants, socks, gloves, and scarves; (3) household item expenditures, such as durable goods, household goods, and household services; (4) transportation and communication expenses, such as vehicle purchases and maintenance, transportation expenses, communication tools, and postal and telephone bills; (5) cultural, educational, and entertainment expenses, such as various types of education expenses, cultural and recreational expenses, and books and newspaper expenses; (6) resident expenditures, such as housing construction, house purchase, rent, water, electricity, and fuel; and (7) other expenses. The statistics for these consumption expenditures were all annual data.

#### Control Variables

In this paper, when using CHE in the regress analysis with household consumption expenditures, other control variables were added. Household income is the total annual income of the family members living together; the market value of the house corresponds to “the current total market price of residential real estate” in the CFPS questionnaire; household non-property assets includes durable goods, productive fixed assets, the sum of each financial asset, and other types of assets; household size is the number of family members living together; household registration (Hukou) is a dummy variable, for which urban household registration is set at 1 and rural household registration is set at 0; middle school education or above is the percentage of family members with a middle school education or above; and people aged 60 years or older is the percentage of the elderly family members aged 60 years or older in the household. In the measurement model, this paper reports values for various income, assets, and consumption expenditure variables in units of 10,000 yuan. Table 1 shows the definition of each of the aforementioned variables, and Table 2 shows the descriptive statistics of the main variables.

**Table 1:** Definitions of the variables.

**Variable**	**Definition**
CHE	1 = occurrence of CHE, 0 = no occurrence of CHE
Med	Medical expenses
Food	Household food expenses and purchases of snacks, beverages, tobacco and alcohol, etc.
Dress	Clothing, footwear, and hat expenditures
Daily	Household goods expenditures
Trco	Transportation and communication expenditures
Eec	Education and entertainment expenditures
House	Resident expenses
Others	Other expenses
Fincome	Household annual income
Fq	Market value of real estate
Fea	Non-property assets
Fs	Family size
Hukou	1 = Urban, 0 = Rural
Fe	Middle school education or above
Fa	Elderly family members aged 60 years or older in the household
Fdcg	Total value of durable goods
Fm	Total operating assets
Fsa	Deposit assets
Flf	Borrowed money from relatives and friends
Flp	Private loans

**Table 2:** Descriptive statistics of the major variables.

**Variable**	**Average value**	**Standard deviation**	**Minimum value**	**Maximum value**
CHE	0.062	0.241	0	1
Med	0.571	1.577	0	38
Food	1.995	1.893	0	29
Dress	0.316	0.538	0	15
Daily	0.911	3.351	0	153.36
Trco	0.542	0.699	0	12.36
Eec	0.657	1.333	0	30.6
House	1.211	3.170	0	55.4
Others	0.129	0.656	0	50.1
Fincome	8.644	19.416	0	915.88
Fq	49.843	110.353	0	5,000
Fea	9.019	31.551	0	1,050
Fs	4.178	2.060	1	19
Hukou	0.518	0.500	0	1
Fe	0.458	0.323	0	1
Fa	0.264	0.323	0	1
Fdcg	4.494	11.729	0	500
Fm	2.882	62.923	0	5,000
Fsa	3.452	18.478	0	1,000
Flf	0.682	3.317	0	100
Flp	0.111	4.441	0	500

*Expenditures and income are total household expenditures and total income within 1 year, respectively; assets and loans are the total household value; the unit is ¥10,000 (the average USD to RMB exchange rate in 2018 was 6.6174)*.

Table 3 provides the statistics for the mean expenditures of households with and without CHEs. There was a difference in each expenditure among all samples, CHE households, and non-CHE households. The mean expenditure of the CHE households was lower than that of all the samples and was significantly lower than that of the non-CHE households. This conclusion is made only based on the results of a simple mean comparison, but the result may be caused by other factors, such as family characteristics and income, which will be further discussed below.

**Table 3:** Statistics of mean consumption expenditure in the catastrophic health expenditure (CHE) and non-CHE groups.

**Variable**	**All samples** ***N*** **=** **14,137**		**CHE households** ***N*** **=** **871**		**Non-CHE households** ***N*** **=** **13,157**	
	**Mean value**	**Standard deviation**	**Mean value**	**Standard deviation**	**Mean value**	**Standard deviation**
Med	1.995	1.893	0.865	0.962	2.070	1.916
Food	0.316	0.538	0.121	0.263	0.330	0.550
Dress	0.911	3.351	0.205	0.516	0.963	3.464
Daily	0.542	0.699	0.233	0.370	0.563	0.711
Trco	0.657	1.333	0.175	0.493	0.691	1.367
Eec	1.211	3.170	0.419	0.554	1.266	3.273
House	0.129	0.656	0.041	0.245	0.135	0.677

### Estimation Method

To explore the effect of CHEs on household consumption, we used propensity score matching (PSM) to estimate the effect of CHEs, i.e., the average treatment effect (ATE). This method, to some extent, overcomes the potential statistical problems, such as self-selection bias and residual variables, related to observation data. PSM was first proposed by Rosenbaum and Rubin (24). It constructs a counterfactual framework that approximates a randomized experiment, so that when comparing the difference between the treatment group and the control group, the dominant bias caused by the observable characteristics can be eliminated to enable the average treatment effect (ATT) when comparing the treatment and control groups.

The treatment group was CHE households, and the control group was non-CHE households. Based on various household characteristic variables, such as annual income, the market value of the real estate, non-property assets, household size, household registration, middle school education or above, and elderly people over 60 years old, we matched households with similar characteristics but differences in the occurrence of CHE. We compared the differences in household consumption to identify the effect of CHEs on household consumption.

First, the propensity score was estimated. Assuming under given control variable *X*, the probability for family *i* in the CHE group is calculated, i.e.,


(1) $$\begin{array}{l} P\left(X\right)\;=\;P_{r}\left(D=1|X\right) \end{array}$$

We used a logit model to obtain the propensity scores for CHE households.


(2) $$\begin{array}{l} P\left(X_{i}\right)=P_{r}\left(D_{i}=1|X_{i}\right)=\exp\left(\beta X_{i}\right)/\left[1+\exp\left(\beta X_{i}\right)\right] \end{array}$$

where *D*_i_ = {0, 1} indicates whether the household is in the treatment group, and *X*_*i*_ represents a series of control variables that may affect the possibility of household i appearing in the treatment group (CHE household or non-CHE household).

Then, PSM was performed. According to the calculated PS value, one-to-one matching was used to find the closest “counterfactual” individual (non-CHE household) for each CHE household.

Finally, based on the matching samples, we calculated the average treatment effect on the treated (ATT). The ATT is the effect of CHEs on household consumption. The equation used to estimate the ATT is


(3) $$\begin{array}{l} ATT=E\left(y_{1i}|D_{i}=1\right)-E\left(y_{0i}|D_{i}=1\right)\\ \;=E\left(y_{1i}-y_{0i}|D_{i}=1\right) \end{array}$$

where *y*_1*i*_ is the household CHE; *y*_0*i*_ is the consumption expenditure of non-CHE households (in the hypothetical state); and *E*(*y*_1*i*_|*D*_*i*_ = 1) is observable, but *E*(*y*_0*i*_|*D*_*i*_ = 1) is not observable and is a counterfactual result, which needs to be replaced by *E*(*y*_0*i*_|*D*_*i*_ = 1) constructed using PSM.

## Empirical Results

The impact of CHEs on various household consumption expenditures was analyzed above using a comparison of means, but the results are susceptible to sample self-selection bias. Therefore, to more clearly analyze the impact of CHEs on various household consumption expenditures, a PSM was used to estimate the ATE of CHEs on each household consumption expenditure, after excluding the effects of other characteristics.

### Logistic Regression of Predicted Propensity Values

Based on the PSM method, we first estimated the probability for the respondent families to become the treatment group (CHE group). The controlling variables in the probability model included household characteristics (e.g., annual household income, market value of real estate, non-property assets, household size, household registration, family members with a middle school education or above, and elderly people over 60 years old in the family) and were matched based on probability.

The logistic regression analysis was performed using household characteristics as explanatory variables and whether the family has CHE as the dependent variable. As shown in Table 4, household income, household real estate market value, household non-property assets, household registration, the proportion of family members with an education of middle school or above, and the proportion of family members aged over 60 years were statistically significant, indicating that selection bias has a certain impact. Therefore, when modeling the effect on household consumption expenditures, we should pay attention to the importance of the selection mechanism.

**Table 4:** Balance test results of matching variables.

**Variables**	**Coefficient**	**Standard error**	***z*-value**	***p*-value**
Fincome	−0.059***	−5.968	0.000	−0.079
Fq	−0.001**	−0.237	0.813	−0.001
Fea	−0.026***	−4.550	0.000	−0.037
Fs	0.014	0.677	0.498	−0.027
Hukou	−0.408***	−4.896	0.000	−0.572
Fe	−0.108*	−0.810	0.418	−0.368
Fa	1.541***	12.822	0.000	1.305
Constant	−2.590***	−18.064	0.000	−2.871

***p < 0.01, **p < 0.05, and *p < 0.1.

### Balance Test

In this section, to examine whether the matching samples that were constructed based on the estimated propensity index through the logistic model described above are in agreement with the balance property, we performed the balance test on the entire sample using the pairwise matching method. Table 5 provides the results of the balance diagnostics for matching variables. The balance diagnostics required no significant differences in the variables between the control group and the treatment group after matching. Some joint hypothesis test results are provided. After matching, the differences in household characteristics between the CHE and non-CHE households were eliminated, and the household differences were controlled within 10%, which enhanced the comparability of the two groups. Meanwhile, the *t*-test result of the balance test on the entire sample indicates that the means of the treatment and control groups are not systematically different, suggesting that the matching is good.

**Table 5:** Balance diagnostics of matching variables.

**Variable**		**Mean value**		**Standard deviation (%)**	**Error reduction (%)**	***T*** **-test**	
		**Treatment group**	**Control group**			***T-*value**	***P-*value**
Fincome	Unmatched	4.340	8.846	−32.8		−6.89	0.000
	Matched	4.340	4.541	−1.5	95.5	−0.74	0.461
Fq	Unmatched	29.094	50.322	−16.2		−5.29	0.000
	Matched	29.094	28.464	0.5	97	0.11	0.911
Fea	Unmatched	2.877	9.625	−27.8		−5.82	0.000
	Matched	2.877	3.125	−1.0	96.3	−0.57	0.571
Fs	Unmatched	3.779	4.192	−19.9		−5.60	0.000
	Matched	3.779	3.697	4.0	80.1	0.77	0.442
Hukou	Unmatched	0.346	0.524	−36.6		−9.89	0.000
	Matched	0.346	0.363	−3.5	90.4	−0.72	0.469
Fe	Unmatched	0.332	0.466	−42.2		−11.55	0.000
	Matched	0.332	0.296	8.2	73.4	0.28	0.023
Fa	Unmatched	0.474	0.252	61.8		19.18	0.000
	Matched	0.474	0.485	−3.0	95.1	−0.55	0.583

### Empirical Results and Analysis

After matching, the difference between the CHE households and the non-CHE households was calculated based on the matching sample population, i.e., the ATT of the treated group (Table 6). Table 6 shows the ATT of CHEs for household consumption expenditures according to equation (3). After matching to eliminate differences in the household characteristics, using CHE > 40%, the average household consumption expenditure of the CHE households was significantly lower than that of the non-CHE households. This result indicates that the CHE households may respond to excess healthcare expenses by reducing various household consumption expenses. This result is similar to the findings by Kim and Yang (17) and Wang et al. (15). Kim and Yang's research also used 40% as the CHE threshold. In addition, this paper extended the CHE threshold to 10–20 and 21–40%. The results showed that CHEs still had a significant negative impact on various household consumption expenditures. Based on the comparison results, the increase in the CHE threshold, the difference in the consumption of various items, such as food expenditures, clothing, footwear, and hats expenditures, cultural, educational, and entertainment expenditures, and resident expenditures, between CHE households and non-CHE households also expanded. This result indicates that CHEs may lead to a continuous decline in the household consumption of non-durable goods related to quality of life, such as food and clothing, and have an important impact on the quality of life of families, i.e., a continuous decline in quality of life.

**Table 6:** The average treatment effect (ATT) of CHEs for various household consumption expenditures.

**Consumption expenditure**	**>40%**			
	**ATT**	**S.E**.	**CHE households**	**Non-CHE households**
Med	−0.557***	0.062	815	11,898
Food	−0.076***	0.016	815	11,898
Dress	−0.389**	0.180	815	11,898
Daily	−0.121***	0.023	815	11,898
Trco	−0.212***	0.036	815	11,898
Eec	−0.478***	0.091	815	11,898
House	−0.037**	0.016	815	11,898
	**21–40%**			
	**ATT**	**S.E**.	**CHE households**	**Non-CHE households**
Med	−0.484***	0.061	1,202	10,696
Food	−0.071***	0.014	1,202	10,696
Dress	−0.533***	0.173	1,202	10,696
Daily	−0.128***	0.023	1,202	10,696
Trco	−0.124***	0.038	1,202	10,696
Eec	−0.453***	0.088	1,202	10,696
House	0.040**	0.019	1,202	10,696
	**10–20%**			
	**ATT**	**S.E**.	**CHE households**	**Non-CHE households**
Med	−0.432***	0.072	1,698	8,998
Food	−0.070***	0.017	1,698	8,998
Dress	−0.547***	0.090	1,698	8,998
Daily	−0.124***	0.026	1,698	8,998
Trco	−0.139***	0.042	1,698	8,998
Eec	−0.389***	0.093	1,698	8,998
House	−0.030**	0.014	1,698	8,998

*, **, and ***represent significant differences at the 10, 5, and 1% levels, respectively.

On the basis of completing a thorough analysis of the impact of CHEs on various household consumption expenditures, we analyzed whether CHEs affect household property and debt and thus analyzed the impact of CHEs on the financial situation of households. To analyze this issue, we combined responses in the CFPS questionnaire at set household asset measurement indicators as total durable goods, total operating assets, and deposit assets (deposits, various types of financial assets, vehicles, etc.) and debt, which included borrowing money from relatives and friends and taking private loans. Table 7 shows the ATT of CHE for household property and debt. When the CHE threshold was set to be higher than 40%, the total value of durable goods and deposit assets of CHE households significantly decreased; however, there was a significant increase in family members borrowing money from relatives and friends, i.e., the occurrence of CHEs reduced household assets and increased debt. Moreover, this effect was different using different CHE thresholds, indicating that as the CHE threshold increased, the number of durable goods decreased when households faced increased medical expenditures, and they used deposit assets to cope with the medical expenditures of a family; however, the operating assets were not significantly affected. The ATT of CHE for borrowing money from relatives and friends also increased significantly with the increase in threshold value, indicating that the increase in household medical expenditures led to a continuous increase in household debt.

**Table 7:** The ATT of CHE on household property and debt.

**Property and debt**	**>40%**			
	**ATT**	**S.E**.	**CHE households**	**Non-CHE households**
Fdcg	−0.743**	0.353	815	11,898
Fm	−5.420	6.144	815	11,898
Fsa	−3.680*	0.225	815	11,898
Flf	0.526***	0.183	815	11,898
Flp	0.085	0.112	815	11,898
	**21–40%**			
	**ATT**	**S.E**.	**CHE households**	**Non-CHE households**
Fdcg	−0.987***	0.310	1,202	10,696
Fm	−2.044	0.374	1,202	10,696
Fsa	−0.184	0.146	1,202	10,696
Flf	0.080*	0.021	1,202	10,696
Flp	0.095	0.182	1,202	10,696
	**10–20%**			
	**ATT**	**S.E**.	**CHE households**	**Non-CHE households**
Fdcg	−1.186***	0.284	1,698	8,998
Fm	−1.446	0.436	1,698	8,998
Fsa	−0.067	0.101	1,698	8,998
Flf	0.067*	0.036	1,698	8,998
Flp	0.082	0.065	1,698	8,998

*, **, and ***represent significant differences at the 10, 5, and 1% levels, respectively.

## Conclusion

The data from the CFPS in 2018 were used for the analyses in this paper. On the basis of considering selectivity bias and heterogeneity, we applied PSM to estimate the effect of CHEs on household consumption expenditures. When the CHE threshold was set to >40%, the occurrence of CHEs had a significant negative impact on other household consumption. If the CHE threshold was expanded, when the CHE threshold was 10–20 or 21–40%, the effect of CHE on the household consumption expenditures still existed. In addition, with the increase in the threshold value, the impact of CHEs on household basic consumption, such as food and clothing, continued to increase, indicating that CHEs impact the basic quality of life of families. Moreover, CHEs not only affected the household consumption expenditure structure but also led to an increase in medical expenditures and household debt and to a reduction in household assets.

Over the years, the Chinese government has been committed to deepening healthcare reform and has gradually established a social medical insurance system that covers all the population, reducing the impact of diseases on individuals and families. However, due to the large population and sporadic and persistent health effects, the objective of this policy has not been fully realized. With the increase in population aging and the slowdown of economic growth in China, the adverse consequences resulting from an increase in household medical costs may continue to worsen. The results of this study indicated that medical costs are still a major problem faced by Chinese families. CHEs not only cause reductions in necessary consumption but may also lead to worsening financial situations for families. Therefore, it is extremely urgent to address the issue of high medical expenses. This study intended to provide evidence for preventing CHE occurrence, reducing the household burden of medical expenses caused by hospitalization, chronic diseases, etc. and, thus, controlling the medical service costs and prompting the development of social medical insurance and other related health policies (15).

Accordingly, the policy implications of this study are as follows. First, studies in the United States and Korea showed that if the actual situation of poor families is not considered, implementing a medical insurance system alone will not solve the burdensome medical expenditures of poor families (17, 25). The Chinese government should regularly track and investigate the incidence of household CHEs to monitor the strength of social medical insurance coverage for individual health risks. Second, governments should continue to improve the social medical insurance system, in particular, increase the popularity of critical illness insurance and commercial medical insurance, increase the level of coverage for individual medical insurance, reduce the incidence of household CHEs, and prevent a large gap in household consumption due to the occurrence of CHEs. Third, medical system reform needs to be further promoted to ensure adequate medical resources, to implement the separation of prescribing and dispensing, and to ensure the rationality of prices for medical services, medicines, and medical equipment. Finally, medical environments should be improved to promote the health status of residents. By providing good medical environments, the confidence of residents in physical and occupational rehabilitation will increase, incomes will stabilize, and the possible impacts of diseases will be minimized.

## Data Availability Statement

Publicly available datasets were analyzed in this study. This data can be found here: http://www.isss.pku.edu.cn/cfps/.

## Author Contributions

NW and WH conducted the data analysis and wrote the first draft of the paper. L-lZ critically reviewed the paper and edited the manuscript. All authors read and approved the final manuscript, involved in planning of the study, and interpretation of results.

## Funding

This study was supported by MOE (Ministry of Education in China) Project of Humanities and Social Sciences (Project No. 17YJC840041).

## Conflict of Interest

The authors declare that the research was conducted in the absence of any commercial or financial relationships that could be construed as a potential conflict of interest.

## Publisher's Note

All claims expressed in this article are solely those of the authors and do not necessarily represent those of their affiliated organizations, or those of the publisher, the editors and the reviewers. Any product that may be evaluated in this article, or claim that may be made by its manufacturer, is not guaranteed or endorsed by the publisher.
